# The Evaluation of Dihydropyrimidine Dehydrogenase Enzyme Level in the Serum of Colorectal Cancer Iraqi Males on Fluoropyrimidine-Based Chemotherapy (Capecitabine)

**DOI:** 10.7759/cureus.44534

**Published:** 2023-09-01

**Authors:** Muhtada A Challoob, Nawar S Mohammed

**Affiliations:** 1 College of Pharmacy, University of Misan, Amarah, IRQ; 2 Department of Biochemistry, College of Medicine, University of Baghdad, Baghdad, IRQ

**Keywords:** 5-fu, 5-fluorouracil, dpd, capecitabine, crc, anticancer

## Abstract

The cornerstone of systemic chemotherapy for colorectal cancer (CRC) revolves around fluoropyrimidines. This class encompasses 5-fluorouracil (5-FU), which is administered intravenously, along with its oral prodrug counterpart, capecitabine. Central to the metabolism of both 5-FU and capecitabine is the pivotal enzyme dihydropyrimidine dehydrogenase (DPD). Operating at the rate-limiting juncture, DPD assumes a critical role. Notably, a deficiency in DPD significantly elevates the risk quotient for encountering unfavorable outcomes linked to the administration of fluoropyrimidines.

This study seeks to assess the significance of DPD enzyme levels in the serum of Iraqi colorectal cancer male patients undergoing fluoropyrimidine-based chemotherapy, specifically with capecitabine. It adopts a case-control design and comprises 80 male participants. Those males are divided into two distinct groups. Group 1 comprises 45 male patients diagnosed with CRC who have experienced relapse subsequent to undergoing chemotherapy based on fluoropyrimidine (capecitabine). Their ages span from 41 to 71 years, and they were treated at the Misan Health Directorate/Misan Center for Tumor Treatment. Group 2 encompasses 35 male patients diagnosed with CRC who underwent fluoropyrimidine-based chemotherapy (capecitabine) without encountering relapse. Their ages range from 40 to 57 years. All participants were provided with comprehensive information regarding the research, and data collection occurred through a structured questionnaire.

Subsequent to capecitabine-based treatment, serum samples were collected from CRC patients (stage III). The findings from this research indicate a notable elevation in DPD enzyme activity. Furthermore, a significant reduction in enzyme activity was observed among patients who experienced relapse, in contrast to those who remained non-relapsed. The results indicate that individuals with an insufficiency in DPD are notably more vulnerable to experiencing severe and potentially life-threatening side effects upon exposure to the commonly utilized chemotherapy drug, 5-FU.

## Introduction

In Iraq, colorectal cancer (CRC) is the fourth most prevalent malignancy and the fifth leading cause of cancer-related mortality [1-3]; moreover, CRC is one of the most dangerous malignancies, spreading to the liver, lungs, and other parts of the gastrointestinal system [4].

The use of fluoropyrimidines is fundamental in the administration of systemic chemotherapy for patients with CRC [5]. Currently, patients with CRC are treated with fluoropyrimidines (5-fluorouracil (5-FU)) intravenously or its oral prodrug capecitabine [6]. Roughly 33% of individuals diagnosed with CRC encounter grade III or more severe toxicities linked to the use of fluoropyrimidines. Additionally, the death rate resulting from this treatment is estimated to be around 0.5% [7,8].

Dihydropyrimidine dehydrogenase (DPD) is an essential enzyme that plays a crucial role in a phase that restricts the rate of metabolism of 5-FU and capecitabine [9]. This route is responsible for the metabolism of about 80% of the given fluoropyrimidines [10]. According to the cited source [11], a small fraction of less than 2% undergoes metabolism via anabolic pathways, resulting in the manifestation of cytotoxic properties specifically targeting tumor cells. The most significant risk factor for the occurrence of adverse events connected to fluoropyrimidine is impairment in dihydropyrimidine dehydrogenase (DPD) [12,13]. A notable observation in individuals with DPD deficiency is a shift toward the generation of active metabolites [14]. Various phenotyping and genotyping methodologies have been devised to identify individuals with dihydropyrimidine dehydrogenase (DPD) deficiency [15].

The deficit of DPD is inherited in an autosomal recessive manner [16]. Individuals who possess two copies of mutant alleles are classified as homozygous for DPD deficiency [17]. Individuals with a pronounced deficit in DPD have notable symptoms that often emerge during early infancy, such as seizures, hypertonia, and mental retardation [18-20]. This type of chemotherapy is linked with potential toxicities such as emesis, diarrhea, mucositis, myelosuppression, and palmar-plantar erythrodysesthesia. The presence of genetic mutations in the enzymes responsible for metabolic processes in cancer patients requiring chemotherapy results in the buildup of metabolites inside the body, particularly in the liver. Consequently, this accumulation leads to hepatotoxicity and neutropenia [21]. A colorectal cancer patient with capecitabine-induced severe toxicity and DPD deficiency was successfully treated with low-dose 5-FU [22].

The aim of this study was to evaluate the importance of serum dihydropyrimidine dehydrogenase (DPD) enzyme levels in Iraqi male individuals who have been diagnosed with colorectal cancer and are currently receiving fluoropyrimidine-based chemotherapy, specifically capecitabine.

## Materials and methods

This study is a case-control design, enrolling participants who were patients under the care of the Misan Health Directorate/Misan Center for Tumor Treatment, located in Misan, Iraq. The research was carried out from November 2022 to April 2023, encompassing a cohort of 80 male participants from Iraq who had previously undergone surgical intervention for stage III colorectal cancer. According to their subsequent surgical treatment, they were divided into two distinct groups. The first cohort consisted of 45 males, aged between 41 and 71 years. These participants encountered a relapse despite receiving adjuvant therapy in the form of a singular cycle of fluoropyrimidine-based chemotherapy (specifically capecitabine). The second cohort comprised 35 male patients with colorectal cancer. Their ages ranged from 40 to 57 years, and these participants had not encountered relapse post-adjuvant therapy. The therapy encompassed a solitary round of fluoropyrimidine-based chemotherapy involving capecitabine.

Patient categorization in cases of relapse was established through the assessment of white blood cell (WBC) counts. The participants received detailed information about the research goals, and data compilation occurred via an extensive questionnaire. Notably, the study strictly adhered to ethical protocols endorsed by the Ethics Committee of the College of Medicine at the University of Baghdad (IRB number: 2023/136A).

The estimation of body mass index (BMI) was conducted in units of kg/m^2^, wherein the calculation included dividing an individual's weight in kilograms by the square of their height in meters. All individuals were measured without footwear using a standardized analog weighing scale. Sera samples were collected in gel tubes from colorectal cancer patients; 5 mL of blood were withdrawn from the same individual in a serum tube and then centrifuged at 4,000 rpm for 10 minutes. The sera samples were collected and stored at -20°C frozen to be used for the determination of the different studied parameters.

Hematology parameters are performed using the GENEX device (GENEX Laboratories, NY, USA), a fully automated hematology analyzer, to rapidly assay whole blood specimens for WBC counts. Alanine aminotransferase (ALT) activity and aspartate aminotransferase (AST) activity in serum were estimated according to the method described by Murray and Kaplan, the alkaline phosphatase (ALP) activity in serum was estimated according to the method described by Wenger, and the albumin concentration in serum was estimated according to the method described by Doumas. All these methods use kits manufactured by Spinreact (Ctra. Santa Coloma, Spain). The evaluation of dihydropyrimidine dehydrogenase activity was conducted using the enzyme-linked immunosorbent assay (ELISA) technique, specifically the dihydropyrimidine dehydrogenase (DPD) ELISA kit, which is designed for in vitro diagnostic purposes, made in Shanghai, China.

Exclusion criteria

Excluded from this study were individuals with a history of tumors in other organs, liver disease, ongoing inflammatory conditions, Cushing's disease, chronic pancreatitis, acromegaly, chronic renal failure, prior pancreatectomy, and both chronic or acute inflammatory disease. Additionally, participants currently using medications, with a smoking history, undergoing lipid-lowering therapy, or engaging in alcohol consumption were also not included.

Statistical analyses

The data gathered underwent analysis through Statistical Package for the Social Sciences (SPSS) software version 23 (IBM SPSS Statistics, Armonk, NY, USA). The analysis encompassed key descriptive metrics: mean, standard deviation (SD), and correlation within the two categories. Significance was established for correlations (measured by coefficient "r"), where p-values were ≤0.05.

## Results

The data presented in Table 1 exhibit a notably substantial decline in white blood cell (WBC) count among relapsed patients, in stark contrast to the non-relapsed group (p < 0.001). Furthermore, the results highlight a statistically significant surge in alanine aminotransferase (ALT) levels within the bloodstream, along with a substantial reduction in serum albumin concentrations among relapsed patients in comparison to their non-relapsed counterparts (p < 0.001). Moreover, the results also highlight a significant rise in aspartate aminotransferase (AST) and alkaline phosphatase (ALP) levels in the serum of relapsed patients when compared with those of non-relapsed patients (p < 0.05). This data collectively emphasizes the marked physiological distinctions between the two patient groups, underscoring the intricate biochemical alterations associated with relapse.

**Table 1:** Correlation between the individual parameters of patients with relapse and without relapse BMI: body mass index, WBC: white blood cells, ALT: alanine aminotransferase, AST: aspartate aminotransferase, ALP: alkaline phosphatase, DPD: dihydropyrimidine dehydrogenase, SD: standard deviation *Statistically significant **Statistically highly significant

Patient parameters	Patients with relapsed (N=45)	Patients without relapse (N=35)	p-value
	Mean ± SD	Mean ± SD	
Age (years)	56.13 ± 9.03	48.00 ± 5.12	0.061
BMI (kg/m^2^)	20.40 ± 2.82	19.29 ± 2.33	0.052
WBC (10^9^/L)	2.247 ± 0.253	3.842 ± 2.251	0.001**
ALT (IU/mL)	51.16 ± 6.30	22.17 ± 4.49	0.002**
AST (IU/mL)	44.73 ± 7.32	36.40 ± 6.44	0.01*
ALP (IU/mL)	152.04 ± 12.16	119.97 ± 8.59	0.02*
Albumin (g/mL)	3.68 ± 0.57	4.61 ± 0.28	0.000**
DPD (IU/mL)	20.96 ± 8.00	75.27 ± 6.86	0.000**

**Figure 1:**
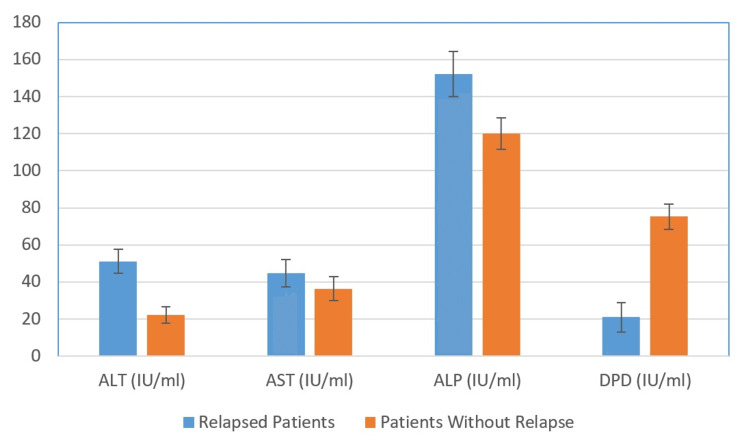
Variations of the mean ± SD of ALT, AST, ALP, and DPD for patients with relapse and patients without relapse ALT: alanine aminotransferase, AST: aspartate aminotransferase, ALP: alkaline phosphatase, DPD: dihydropyrimidine dehydrogenase, SD: standard deviation

The data presented in Table 1 highlight a noticeable reduction in serum DPD activity among patients experiencing relapse, in stark contrast to those patients who remained relapse-free (p < 0.001). This significant difference in DPD activity between the two groups signifies a noteworthy finding. Figure 1 shows the variations of the mean ± SD of ALT, AST, ALP, and DPD for patients with relapse and those without.

Table 2 illustrates a noteworthy and statistically significant positive correlation between the concentration of serum albumin and DPD enzyme activity (p < 0.05). Conversely, a significant negative correlation is observed between serum DPD enzyme activity and the enzyme activities of AST and ALT (p < 0.05). Additionally, our findings indicate the absence of a significant correlation between serum ALP enzyme activity and DPD enzyme activity.

**Table 2:** Correlation between DPD and liver biomarkers ALT: alanine aminotransferase, ALP: alkaline phosphatase, AST: aspartate aminotransferase, DPD: dihydropyrimidine dehydrogenase *Statistically significant

Biomarker	Correlation	p-value
ALT (IU/mL)	-0.134	0.018*
Albumin (g/mL)	0.121	0.015*
ALP (IU/mL)	-0.303	0.092
AST (IU/mL)	-0.164	0.027*

The results from this study's phenotyping analysis indicate that individuals who experienced a relapse and were administered oral capecitabine exhibited reduced DPD enzyme levels in their bloodstream.

## Discussion

The rate-limiting enzyme involved in capecitabine metabolism is DPD enzyme, which converts 5-FU to the much less toxic form known as 5-fluoro-5,6-dihydro-fluorouracil to 5-fluoro-ureido-propionic acid, and finally the ultimate catabolites, mainly α-fluoro-β-alanine; therefore, any genetic defect in this enzyme leads to hepatotoxicity because of the accumulation of its metabolites in the liver [23,24]. Fluorouracil is associated with mitochondrial membrane collapse and a reduction in membrane potential that might impair the oxidation of fatty acids and lead to the subsequent accumulation of reactive oxygen species (ROS) within hepatocytes [25]. In addition, the metabolic breakdown of fluorouracil yields catabolites, including fluoro-β-alanine, which has the potential to impair the hepatocytes' ability to metabolize various substances, including medicines and lipids [26]. It is believed that the mechanism of chemotherapy-induced hepatotoxicity is secondary to the production of reactive oxygen species (ROS), which is intended to induce tumor cell apoptosis [26].

It is widely acknowledged that genetic factors play a significant role in the susceptibility to 5-FU-induced severe toxicity. Specifically, variations in the functionality of the dihydropyrimidine dehydrogenase enzyme have been identified as a crucial component in determining the risk of toxicity [27]. To date, more than 160 distinct allele variations, also known as single nucleotide polymorphisms (SNPs), have been discovered for the dihydropyrimidine dehydrogenase (DPD) enzyme, which has an impact on its enzymatic function. According to estimates, the prevalence of total dihydropyrimidine dehydrogenase (DPD) deficiency is around 0.1%-0.01% among persons, whereas a partial DPD deficiency is seen in approximately 3%-5% of the population. Nevertheless, there are variations in the occurrence of pathogenic DPD mutations across different countries and ethnic populations [28].

Our findings agree with the study done by Melliti et al., who studied the DPD activity in the serum of Tunisian patients with partial deficiency of DPD after treatment with 5-FU-based chemotherapy [29]. Also, our results agree with van Kuilenburg et al., who studied the phenotype of variants in the dihydropyrimidine dehydrogenase gene in healthy Dutch volunteers [30].

Limitations

This research possesses certain constraints, notably being a single-center study, consequently leading to a relatively limited sample size.

## Conclusions

The findings of this study reveal that the administration of capecitabine results in hepatocyte injury, leading to elevated liver enzyme levels in patients' bloodstreams. This adverse effect is attributed to an inadequate level of the enzyme DPD, which is closely associated with heightened susceptibility to severe and potentially life-threatening complications upon exposure to the widely employed chemotherapy agent, 5-FU. The integration of this knowledge into clinical practice can significantly aid in selecting the most suitable oncological treatment approach.
